# DYNAMIC INTERPLAY BETWEEN STRESS AND POOR SLEEP AND THEIR CONSEQUENCES ON DAILY WELL-BEING

**DOI:** 10.1093/geroni/igad104.1922

**Published:** 2023-12-21

**Authors:** Soomi Lee, David Almeida

## Abstract

Stress impairs sleep. Poor sleep also increases the likelihood of stressful experiences. Importantly, there is the vicious cycle between stress and poor sleep, such that having either one increases the odds of the other. This is problematic in the context of aging, because midlife and older adults may have physiological vulnerability to stressors and age-related deterioration in sleep. This stalemate wherein aging individuals are exposed to daily stressors and degraded sleep but need to lower stress and improve sleep for long-term health calls for further research describing how stress and poor sleep co-occur in daily life and contribute to daily health and well-being. To respond to this call, this symposium brings together four rigorous studies. Paper 1 examines how recession hardships (financial, job, housing-related) are associated with daily sleep quality in midlife and older adults. Paper 2 focuses on exposure and reactivity to daily interpersonal stressors and reports their associations with daily sleep quality and physical activity in older adults. Paper 3 investigates daily links between worry and rumination and their implications for sleep in older adults. Paper 4 examines how consecutive poor sleep is associated with day-to-day trajectories of rumination and memory lapses in midlife and older adults. These papers use relevant theories and advanced analytic techniques to describe the nature, causes, and consequences of stress and poor sleep in the second half of life. The discussant, Dr. David Almeida will integrate key findings from these studies, discuss their theoretical and methodological contributions, and consider opportunities for future research.

